# Editorial: Rho family GTPases and their effectors in neuronal survival and neurodegeneration

**DOI:** 10.3389/fncel.2023.1161072

**Published:** 2023-02-21

**Authors:** Daniel A. Linseman, Qun Lu

**Affiliations:** ^1^Department of Biological Sciences, Knoebel Institute for Healthy Aging, University of Denver, Denver, CO, United States; ^2^The Harriet and John Wooten Laboratory for Alzheimer's and Neurodegenerative Diseases Research, Department of Anatomy and Cell Biology, Brody School of Medicine, East Carolina University, Greenville, NC, United States

**Keywords:** Rho, Rho kinase (ROCK), Rac (Rac GTPase), cell division cycle 42 GTP-binding protein (Cdc42), neurodegeneration, neurodegenerative disease

In addition to their prominent roles in regulating actin cytoskeletal dynamics and neuronal morphology, Rho family GTPases are significant players in neuronal survival and death. In this Research Topic, we highlight Rho family GTPase-dependent pathways that contribute to pathogenic mechanisms of neurodegenerative disorders. A thorough investigation of these topics reveals diverse regulators and effectors of Rho GTPase function that provide novel strategies to target Rho family GTPase signaling pathways for therapeutic intervention in neurodegeneration.

In the current Topics Issue, Guiler et al. review several classes of pharmacological modulators of Rho GTPase activity and discuss their potential utility in the treatment of neurodegenerative disorders. Notable examples include the inhibition of Rho/Rho kinase (ROCK) activity in Alzheimer's disease (AD) using small molecules like fasudil or natural products such as *Xanthoceras sorbifolia* extract, therapeutic effects of ROCK inhibitors in mouse models of amyotrophic lateral sclerosis (ALS) and spinal muscular atrophy, and enhanced autophagic clearance of alpha-synuclein and increased dopaminergic neuron survival in Parkinson's disease following activation of Rac1. It is evident that modulating the function of Rho GTPases or their downstream effectors has significant translational potential in diverse neurodegenerative disorders. However, to realize the full therapeutic potential of Rho GTPase modulators, one needs to clarify the intricate regulation of these G-proteins and reveal how multiple family members work in concert to impact disease progression. The additional articles in this Topics Issue focus on downstream effectors and upstream regulators of Rho GTPase signaling, as well as Rho family crosstalk with other small GTPase families, within the broader context of neurodegeneration.

Rho/ROCK signaling has been implicated as a pathogenic pathway in several neurodegenerative disorders. In AD, Rho activation induced by amyloid-beta (Aβ) causes dendritic spine degeneration *via* a ROCK2-dependent mechanism, suggesting that inhibition of ROCK2, or it's downstream target LIMK1, may preserve synapse structure in AD (Henderson et al., 2019). Intriguingly, inhibition of ROCK2 may have additional therapeutic benefits in AD. In the current Topics Issue, Weber and Herskowitz highlight the potential role of ROCK2 in regulating the autophagic clearance of pathological Tau protein. Together, the capacity of ROCK inhibitors to preserve synapse architecture while also stimulating the autophagic clearance of pathological Tau suggest that compounds targeting this kinase family and more specifically ROCK2, may have significant therapeutic benefit in AD.

Rho GTPases are central regulators of the actin cytoskeleton and they may also contribute to neurodegeneration *via* this most basic function. In the current Topics Issue, Wurz et al. discuss the role of cofilin-actin rods in neurodegeneration. Cofilin-actin rods are formed in neurons undergoing stress and appear to be pathogenic players in early-stage neurodegeneration. A major component of cofilin-actin rods is dephosphorylated cofilin. In AD, cofilin is dephosphorylated following the Aβ-induced activation of Cdc42 and corresponding downregulation of RhoA and the cofilin kinase LIMK (Davis et al., 2009; Chen and Wang, 2015). Studies performed in AD model mice indicate that overexpression of LIMK1 increases the phosphorylation of cofilin and enhances memory formation (Zhang et al., 2021). These studies provide further evidence that the Rho GTPase downstream effector, LIMK, plays a significant role in neurodegeneration.

In addition to downstream effectors like ROCK and LIMK, upstream regulators of Rho GTPases have also been implicated in neurodegeneration. One interesting regulator of Rho/ROCK function is the cellular prion protein (PrPC). Kim et al. (2017) showed that the PrPC facilitates an interaction between RhoA and p190RhoGAP, leading to the inactivation of Rho/ROCK signaling and enhancement of neurite outgrowth. In contrast, disease-associated mutations in PrPC impair RhoA inactivation which contributes to prion-mediated neurodegeneration. In the current Topics Issue, Schneider et al. discuss the potential consequences of the dysregulation of PrPC influence on Rho/ROCK, and how the resulting aberrant Rho/ROCK signaling enhances neuronal sensitivity to neuroinflammation while also amplifying the production of neurotoxic amyloid species.

Another distinct upstream regulator of Rho GTPases is delta-catenin, a PDZ domain binding protein enriched in the postsynaptic density. In the current Topics Issue, this relationship is detailed by Donta et al.. Delta-catenin promotes the formation of dendritic spines and arborization of the dendritic tree (Abu-Elneel et al., 2008; Yuan et al., 2015). It mediates these effects in part, through regulation of Rho GTPase activity. Delta-catenin inhibits RhoA through the sequestration of p190RhoGEF (Kim et al., 2008). In contrast, delta-catenin increases Rac1 and Cdc42 activity and this effect is required for increasing dendritic spine density (Abu-Elneel et al., 2008). Intriguingly, multiple mutations in delta-catenin are associated with AD (Jun et al., 2012). This finding has led to speculation that loss of function mutations in delta-catenin may contribute to synapse degeneration in AD *via* the aberrant activation of Rho GTPase.

In addition to upstream regulators and downstream effectors, crosstalk of Rho GTPases with other small GTPase families may also play a role in neurodegeneration. In the current Topics Issue, Nik Akhtar et al. highlight crosstalk between the Rho and Rab GTPase families. A notable case is that of ALS in which genes mutated in this disease (alsin and C9orf72) are known guanine nucleotide exchange factors (GEFs) for both Rho and Rab family members. Alsin (ALS2) possesses GEF activity for both Rac1 and Rab5 (Topp et al., 2004), while C9orf72 displays GEF activity, at least *in vitro*, for multiple Rab and Rho family members (Iyer et al., 2018). In ALS, Rac1 and Rab5 appear to reciprocally control one another *via* the complex GEF activity of Alsin (Topp et al., 2004). Given that Rho and Rab are central regulators of the actin cytoskeleton and vesicular transport, respectively, crosstalk between these GTPase families influences diverse processes that contribute to neurodegeneration.

In summary, the articles presented in the current Topics Issue highlight the role of downstream effectors and upstream regulators of Rho GTPase function, as well as crosstalk with the Rab GTPase family, in the pathogenesis of neurodegeneration. Further elucidation of the complex interplay of Rho GTPases and their upstream and downstream signaling networks should identify novel targets for therapeutic intervention in neurodegenerative disorders.

## Author contributions

DL prepared the first draft of the manuscript. QL reviewed and edited the final version. All authors contributed to the article and approved the submitted version.
